# Provider Perceptions of the Impact of Rapid Whole Genome Sequencing on Care and Management

**DOI:** 10.21203/rs.3.rs-9173307/v1

**Published:** 2026-04-10

**Authors:** Rachel Palmquist, Chelsea Solorzano, Brian J. Shayota, Dawn Bentley, Carrie Torr, Julie M. Porter, Emily Fleming, LeeAnn Dempsey, Martin Tristani-Firouzi, Joshua L. Bonkowsky, Timothy M. Bahr, Sabrina Malone Jenkins

**Affiliations:** University of Utah; University of Utah; University of Utah; University of Utah; University of Utah; University of Utah; University of Utah; University of Utah; University of Utah; University of Utah; Intermountain Healthcare; University of Utah

**Keywords:** Rapid Genome Sequencing, Neonatal Genetics, Hospital Genetics, Medical Genetics, Provider Survey

## Abstract

**Objectives:**

Rapid whole genome sequencing (rWGS) is emerging as the standard-of-care for critically ill neonates and children. In this study, we surveyed healthcare providers to explore the impact of rWGS on medical decision making, patient management, and communication with families.

**Patients and Methods:**

We designed a survey to measure provider perceived impact of rWGS in critically ill neonates and children sequenced at a single center, Primary Children’s Hospital (Salt Lake City, UT). The survey was completed by the primary critical care provider or primary specialist provider following rWGS result return. Survey results were stratified by result type and unit.

**Results:**

The majority of results (81%, 140/172) were considered ‘Useful’ or ‘Very useful’ and increased clarity for the care team (52%, 89/171) and family (62%, 96/156). Providers identified at least one change in management for the majority of results (57%, 98/171).

**Conclusions:**

This study found positive provider perceptions for rWGS in both pediatric non-ICU and ICU hospitalizations. A majority of providers identified the rWGS results as useful, and impactful for management. Standardized evaluation of the care team’s perception of clinical management at the time of result return is a helpful measure for understanding the clinical utility of rWGS.

**Clinical Trial registry name and registration number:**

No clinical trial registry. This study was approved by the Institutional Review Board (IRB) of the University of Utah (IRB_00131255) as an exempt study. A consent cover letter was utilized and consent to participate was given by completing and returning the questionnaire.

## Introduction

The use of rapid next-generation sequencing (NGS) technologies in pediatric critical care units has been shown in multiple studies to provide earlier diagnosis with positive impacts on patient outcomes and reduced length of stay (Farnaes et al., 2018; Kapil et al., 2019; Kingsmore et al., 2024; Saunders et al., 2012). The impact of rapid whole genome sequencing (rWGS) on changes in medical management of critically ill patients has varied from 30–72% (Dimmock et al., 2020; Kingsmore et al., 2019, 2024; Palmquist et al., 2022; Petrikin et al., 2018). This variation is suspected to be, in part, due to the differences in how clinical management is defined and evaluated, in addition to the wide spectrum of presentations reported on(Callahan et al., 2023). Given the difficulty in identifying control groups for rare genetic diagnoses, studies of rWGS clinical impact have primarily evaluated change in management through Delphi method evaluation, and primarily in ICU settings. NISGHT2, a randomized control study evaluating rapid whole exome sequencing (rWES) and rWGS, surveyed care providers to assess perceived changes in clinical management(Dimmock et al., 2020). Using perceived utility of rWGS as the primary outcome, this study found 77% of patients had results that were considered to be useful. Additionally, change in management was reported in 28% of cases. NSIGHT2 was limited to neonates and infants in ICUs. A number of other studies have also summarized the use of rWGS in the pediatric ICU setting (Bonser et al., 2023; Carey et al., 2020; Rodriguez et al., 2024; Sanford et al., 2019). There is emerging (Keefe et al., 2025), however limited data, on the significant impact of rapid NGS and perception of providers in other pediatric clinical settings.

To explore provider perceived impact of rWGS across all pediatric inpatient settings we developed a survey that was administered to providers at a children’s hospital. Here we characterize survey results describing how rWGS is incorporated into medical decision making, management, and communication with families in ICU settings (neonatal intensive care unit (NICU), pediatric intensive care unit (PICU), cardiac intensive care unit (CICU)), and in non-intensive care units (non-ICU).

## Methods

This study was approved by the Institutional Review Board (IRB) of the University of Utah (IRB_00131255) as an exempt study. A consent cover letter was utilized and consent to participate was given by completing and returning the questionnaire. Study location was Primary Children’s Hospital (PCH), a 289 bed freestanding tertiary care children’s hospital, which serves as the sole tertiary pediatric center for an estimated pediatric population of > 1.7 million children (Bureau, n.d.; U.S. Census Bureau QuickFacts, n.d.). The survey development group included neonatologists, genetic counselors, geneticists, neonatology research nurses and science communication researchers. The questions assessed providers’ perceptions regarding if the genetic result changed the medical care plan, the impact of the genetic result on clinical care and family comprehension, and perceived clinical utility of the test. The survey was built and administered in REDCap with an anticipated completion time of 5–10 minutes.

The REDCap link for survey completion was emailed to care providers of patients who received rWGS while admitted to PCH between July 2020 and April 2023. This survey was sent to the primary intensivist attending or fellow for ICU patients and the primary specialist involved with rWGS ordering for non-ICU patients within 1–5 days following return of laboratory result. Survey requests were emailed to providers a total of three times.

Descriptive analysis describing provider survey responses and comparing response rates by result type and hospital unit was completed. Fisher’s exact test was used to compare responses across sub-groups. Partially completed surveys were included for analysis if one or more sections were completed, therefore the denominator used in calculations varies slightly for overall surveys, usefulness, change in management, and other clinical impacts.

## Results

Surveys were distributed for 258 patients over a 33-month period. Surveys were completed for 173 of 258 patients (67% completion rate); 91% were completed by attending physicians and 9% by subspecialty fellows. NICU providers had the highest survey completion rate at 73% (47/64), followed by CICU at 71% (30/42), non-ICU units at 65% (39/60) and PICU at 62% (57/92). For the surveys completed, 27% (47/173) of patients were in the NICU at time of testing, 33% (57/173) in the PICU, 17% (30/173) in the CICU and 23% (39/173) on other non-ICU floors.

Results were identified by providers as positive in 49% (84/173), uncertain in 20% (35/173), and negative in 31% (54/173) of returned surveys (Table 1, Supplementary Table 1).

Given the diverse ways that genomic testing can inform patient care, providers were asked to broadly evaluate the clinical utility of genomic testing for their patient, from ‘Very Useful’ to ‘Not Useful at All’. The majority of results (81%, 140/172), and all but two positive results (98%, 82/84) were identified as ‘Useful’ or ‘Very useful’, with no results reported as ‘Not at All Useful’. Providers identified at least one change in management resulting from rWGS results for the majority of patients (57%, 98/171). For 15 of 171 surveys, the change in clinical management selected was “other” but “other” was not specified in the comments, and if removed, 50% (86/171) specified one or more types of changes in management. Consistent with prior studies and clinical expectations, patients with positive results were significaantly more likely (p < 0.001) to have a change in management identified (83%, 68/82), however, around a third of patients with uncertain (26%, 9/35) or negative results (39%, 21/54) were also reported to have at least one change in management. The most common changes in management for positive results included referral to a specialist (37%, 30/82), additional medical screening initiated (27%, 22/82), and medical therapy initiated (24%, 20/82). Stopping of a medical therapy and avoidance of a procedure or diagnostic test were reported in 5% (4/82) and 18% (15/82) of positive cases, respectively (Table 2, Supplementary Table 1). Assessing the avoidance of therapies, procedures and tests is important as these have the potential to contribute to the cost-effectiveness of rWGS, although associated costs were not assessed as part of this study.

Providers reported the majority of results provided clarity for the care team (52%, 89/171) and family (62%, 96/156). Cases with results identified as positive had significantly higher reporting (p < .0001) of increased clarity for care team (72%, 60/83) and family (78%, 65/83), however, notably, 41% (22/54) of cases with negative results were reported to increase clarity for providers and for families. A commonly expressed concern is that genetic test results may cause confusion for providers and families, especially for variants of uncertain significance. Overall, providers only reported increased confusion for the care team (3%, 5/171) and family (8%, 12/156) in a minority of cases. While reported in several cases with positive and negative results, the uncertain results contributed the most to increased confusion for the care team in 9% of patients (3/34) and for provider reported increase in confusion for family in 14% of patients (4/28). Of note, increased confusion for the family was noted in 11% (5/45) of negative results, indicating improved communication around negative results and planned follow up may be beneficial (Table 2, Supplementary Table 1).

**Figure 1:**
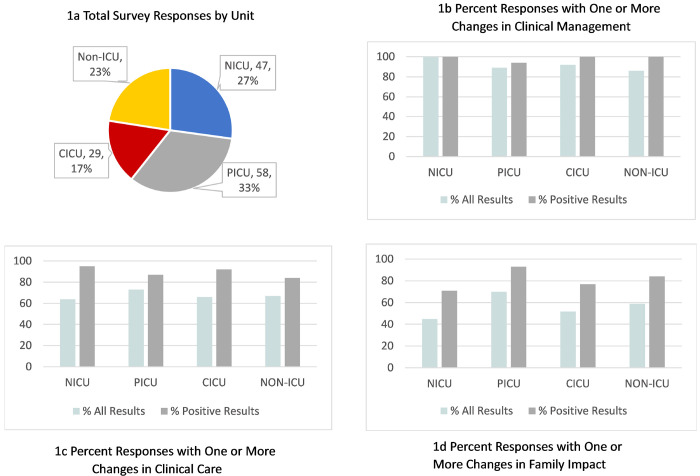
Comparison of Survey Results by Unit at Time of Result Return a) Proportion of total responses by unit; if in multiple units during admittance unit of highest acuity used. Provider reported b) responses with one or more changes in clinical management c) responses with one or more changes in clinical care and d) responses with one or more changes in family impact are stratified by unit and by all results and positive results. The proportion of WGS responses that resulted in impact for all categories of change was not statistically different across units overall or when stratified by results type.

Further subdividing results by hospital unit (Fig. 1, Supplementary Table 1), units that most frequently reported at least one change in management were the PICU (71%, 40/56) and non-ICU units (59%, 23/39), followed by CICU (52%, 15/29) and NICU (45%, 21/47). There was not a significant difference in change in management between units overall (p = .551) or when stratified by positive (p = .917), negative (p = .191) or uncertain (p = .493) results. Notably, the non-ICU units had the most frequent reports of change in management for several impactful categories including initiation of medical therapy with 23% (9/39) of all non-ICU cases and 42% (8/19) of positive non-ICU cases, dietary changes with 8% (3/39) of all non-ICU cases and 16% (3/19) of positive non-ICU cases, and initiation of additional medical screening with 21% (8/39) of all non-ICU cases and 42% (8/19) of positive non-ICU cases. (Fig. 1, Supplementary Table 2).

## Discussion

Our study found that a majority of providers considered the results of rWGS to be useful or very useful, identified at least one change in clinical management and increased clarity for both care team and families in ICU and non-ICU settings. Prior studies have provided substantial evidence regarding the significant rates at which rWGS findings lead to changes in clinical management in the neonatal and pediatric critical care settings (Bonser et al., 2023; Cakici et al., 2020; Carey et al., 2020; Daoud et al., 2016; Dimmock et al., 2020; Farnaes et al., 2018; French et al., 2019; Kingsmore et al., 2019; Palmquist et al., 2022; Rodriguez et al., 2024; Sanford et al., 2019; Willig et al., 2015). This study further supports the value of rWGS in supporting care management decision making in ICU and non-ICU settings. We found that for all units the majority of results were identified as ‘useful’ or ‘very useful’ (81%, 140/172) and were associated with one or more changes in management (57%, 98/171). This is similar to provider perceptions reported through the NSIGHT2 study of neonatal and pediatric ICUs, where 77% of all results were reported as useful (Dimmock et al., 2020). We found that results that the provider identified as ’positive’, were most frequently reported as having an impact on clinical management and care, as well as perceived impact on family. Reported impact on management and care was comparable for patients in the ICU and non-ICU settings (Fig. 1).

Provider responses highlight the importance of rWGS results in providing clarity and improving communication for care providers and families in all units. The impact of results on care team and family clarity were within similar ranges across all units (Fig. 1d). There were several cases where results were reported to increase confusion for the care team and perceived to increase confusion for the family. The report of increased confusion for care team was consistent with NSIGHT2 (Dimmock et al., 2020) (3%), however the perceived increase in confusion for families was higher (8%). Varying levels of confusion, frustration and stress with genetic test results are reported across various specialties, especially with uncertain or negative results in the setting of undiagnosed disease(Clift et al., 2020; Peter et al., 2022). Given this, it is important to assess strategies that minimize confusion, including assessing family perspectives directly. This will be explored as part of an upcoming implementation project for the use of genomic sequencing at Primary Children’s and five other regional hospitals. With limited numbers, there was a notable trend in the way that VUSs and negative cases were reported as influencing clarity in the non-ICU compared to other units. Reports identified as uncertain in non-ICU units were more often considered to improve care team and family clarity as compared to ICU units, whereas negative results were less often considered to improve care team and family clarity in comparison to ICU units. Further evaluation would be needed to determine if this trend is reproducible and if it is related to the setting or the respondent. Non-ICU provider responders were predominantly geneticists and neurologists compared to ICU responders being neonatologists and intensivists, and it is possible the way they incorporate VUS and negative result types into care may influence survey responses.

Limitations of our study include that results were from perceptions of a single provider at the time of result return and confirmation of diagnostic status and clinical changes in management were not separately verified through chart review. Future studies could evaluate perceptions of multiple providers for the same case, as well as compare provider reported management changes to documented actions. Only limited information on the completing provider was collected; future studies could consider assessing if provider characteristics such as specialty, years in practice, or genetics knowledge may influence responses. On review, several questions could benfit from further modification to better capture data for the intended purpose. Results were grouped for analysis by provider identification of result as positive, uncertain, or negative, however this did not always correlate with providers perception that the results were an explanation for symptoms (Table 1). In future studies, we would opt to further refine these questions to improve assessment of provider perceptions around the diagnostic status of results. Two questions were identified that we suspect may have been inconsistently interpreted. The first question inquired if the diagnosis was suspected prior to test, with providers reporting this for 24% (35/151) of all patients and 15% (5/34) of patients with negative results. Anecdotally, based on this infrequently being the case, and with seeing this reported in negative cases, we anticipate it was not clear that ‘diagnosis’ was referencing the specific genetic diagnosis identified. Additionally, only 25% of all patients and 41% of positive reports reported rWGS shortened time to diagnosis. In future studies it may be helpful to clarify a comparison group for this, such as comparing to non-rapid genetic evaluation.

We found that only 4% of patients (6/171) were reported to have shortened length of stay as a management change. The survey was administered shortly after result return, which may have made impact on length of stay difficult to predict or determine. Given that impact on length of stay is one of the primary outcomes that has been utilized for outcome and cost-efficiency studies (Incerti et al., 2022; Sanford Kobayashi et al., 2022), future studies may consider assessing change in length of stay at time of discharge or directing providers to determine if they anticipate shortened length of stay in comparison to similar control group.

Clinical research efforts have established the utility of rWGS for neonates, infants, and children in intensive care units. Our study provides further support of the beneficial impact of rWGS not only in ICUs, but also evidence that rWGS is a useful tool that impacts clinical care management for children hospitalized in lower acuity units. This is an important consideration as a majority of Medicaid and commercial insurers have admittance to an ICU as inclusionary criteria for use of rWGS, and further that many of them only cover infants under one year of age. In our experience, since many children, including those in non-ICU units, have conditions requiring repeat hospitalizations prior to and after testing, an rWGS result even if returned following discharge, is still impactful. To further evaluate the relevance of rWGS in this setting, a chart review of patients receiving rWGS in non-ICU units and the associated documented care management changes is under way at our institution.

## Supplementary Material

This is a list of supplementary files associated with this preprint. Click to download.

SupplementaryTable1Submitted.xlsx

## Figures and Tables

**Table 1:** Provider Perception of Result and Result Utility

Provider Credentials	All	Positive	Uncertain	Negative	P-value

	91% Attending (158/173)	94% Attending (79/84)	86% Attending (30/35)	91% Attending (49/54)	Not meaningful
	9% Fellow (15/173)	6% Fellow (5/84)	14% Fellow (5/35)	9% Fellow (5/54)	
Understanding of patients result	25% Explains ALL of symptoms (42/169)	48% Explains ALL of symptoms (40/83)	3% Explains ALL of symptoms (1/34)	2% Explains ALL of symptoms (1/52)	<0.001
	18% Explains SOME of symptoms (31/169)	33% Explains SOME of symptoms (27/83)	6% Explains SOME of symptoms (2/34)	4% Explains SOME of symptoms (2/52)	
	17% MAY explain symptoms (28/169)	11% MAY explain symptoms (9/83)	56% MAY explain symptoms (19/34)	0% MAY explain symptoms (0/52)	
	40% Does NOT explain symptoms (68/169)	8% Does NOT explain symptoms (7/83)	35% Does NOT explain symptoms (12/34)	94% Does NOT explain symptoms (49/52)	

Diagnosis suspected prior to test	24% Yes (36/151)	35% Yes (29/84)	6% Yes (2/33)	15% Yes (5/34)	<0.001
	55% No (83/151)	52% No (44/84)	52% No (17/33)	65% No (22/34)	
	21% Uncertain (32/151)	13% Uncertain (11/84)	42% Uncertain (14/33)	21% Uncertain (7/34)	

Result returned after discharge	36% Yes (62/170)	32% Yes (26/82)	34% Yes (12/35)	45% Yes (24/53)	0.265
	64% No (108/170)	68% No (56/82)	66% No (23/35)	55% No (29/53)	

Clinical utility of genomic testing	0% Not Useful At All (0/172)	0% Not Useful At All (0/84)	0% Not Useful At All (0/35)	0% Not Useful At All (0/53)	<0.001
	4% Not Very Useful (7/172)	0% Not Very Useful (0/84)	3% Not Very Useful (1/35)	11% Not Very Useful (6/53)	
	15% Neutral (25/172)	2% Neutral (2/84)	29% Neutral (10/35)	25% Neutral (13/53)	
		26% Useful (22/84)	43% Useful (15/35)	42% Useful (22/53)	
	34% Useful (59/172)	72% Very Useful (60/84)	26% Very Useful (9/35)	23% Very Useful (12/53)	
	47% Very Useful (81/172)				

**Table 2:** Provider Perception of Result Impact on Management, Clinical Care and Family

How did rWGS impact clinical management plan?					
	*All*	*Positive*	*Uncertain*	*Negative*	
**Surveys with one or more positive or actionable changes in management selected (excludes 'it did not change management)**	57% (98/171)	83% (68/82)	26% (9/35)	39% (21/54)	< 0.001
*It did not change management*	43% (73/171)	17% (14/82)	74% (26/35)	61% (33/54)	< 0.001
*Medical therapy initiated*	13% (22/171)	24% (20/82)	6% (2/35)	0% (0/54)	< 0.001
*Medical therapy stopped*	2% (4/171)	5% (4/82)	0% (0/35)	0% (0/54)	0.138
*Dietary change*	4% (7/171)	6% (5/82)	3% (1/35)	2% (1/54)	0.605
*Avoidance of procedure/diagnostic test*	14% (24/171)	18% (15/82)	3% (1/35)	15% (8/54)	0.068
*Early tracheostomy and/or gastrostomy tube placement*	1% (2/171)	2% (2/82)	0% (0/35)	0% (0/54)	0.700
*Referral to specialist*	21% (36/171)	37% (30/82)	6% (2/35)	7% (4/54)	< 0.001
*Additional medical screening*	18% (30/171)	27% (22/82)	11% (4/35)	7% (4/54)	0.008
*Palliative care initiated*	5% (8/171)	10% (8/82)	0% (0/35)	0% (0/54)	0.011
*Palliative care withdrawn*	1% (1/171)	1% (1/82)	0% (0/35)	0% (0/54)	> 0.99
*Change in medical care unrelated to primary diagnosis*	6% (10/171)	9% (7/82)	3% (1/35)	4% (2/54)	0.474
*Shortened length of stay*	4% (6/171)	4% (3/82)	0% (0/35)	6% (3/54)	0.408
*Other*	15% (25/171)	15% (12/82)	11% (4/35)	17% (9/54)	0.780
How did rWGS impact clinical care?					
	*All*	*Positive*	*Uncertain*	*Negative*	
**Surveys with one or more positive or actionable impacts on care selected (excludes increased confusion and was not helpful or did not change care)**	**69% (118/171)**	**89% (74/83)**	**44% (15/34)**	**50% (27/54)**	**< 0.001**
*Provided clarity for the primary care team*	52% (89/171)	72% (60/83)	21% (7/34)	41% (22/54)	< 0.001
*Changed management plan for consulting specialist*	26% (45/171)	45% (37/83)	15% (5/34)	6% (3/54)	< 0.001
*Shortened length of time to diagnosis*	25% (42/171)	41% (34/83)	12% (4/34)	7% (4/54)	< 0.001
*Created more confusion for the care team*	3% (5/171)	1% (1/83)	9% (3/34)	2% (1/54)	0.101
*Improved communication of outcome/prognosis with family*	45% (77/171)	67% (56/83)	29% (10/34)	20% (11/54)	< 0.001
*The result was not helpful or did not change care*	30% (52/171)	10% (8/83)	53% (18/34)	48% (26/54)	< 0.001
How did your patients rNGS result affect the family?					
	*All*	*Positive*	*Uncertain*	*Negative*	
**Survey responses with one or more positive or actionable effects on family identified (excludes increased confusion)**	92% (144/156)	99% (82/83)	86% (25/28)	91% (41/45)	0.038
*Provided clarity for the family*	62% (96/156)	78% (65/83)	32% (9/28)	41% (22/45)	< 0.001
*Additional familial screening*	19% (30/156)	27% (22/83)	25% (7/28)	2% (1/45)	< 0.001
*Reproductive counseling/planning*	19% (30/156)	30% (25/83)	18% (5/28)	0% (0/45)	< 0.001
*Family referred to services*	16% (25/156)	27% (22/83)	7% (2/28)	2% (1/45)	< 0.001
*Created more confusion for family*	8% (12/156)	4% (3/83)	14% (4/28)	9% (5/45)	< 0.065
*Other*	23% (36/156)	10% (8/83)	39% (11/28)	31% (17/45)	< 0.001
